# Quantification of Ocular Gene Therapy–Related Inflammation Using the VRMACS Grading System

**DOI:** 10.1016/j.xops.2026.101316

**Published:** 2026-07-06

**Authors:** Maram E.A. Abdalla Elsayed, Robert E. MacLaren

**Affiliations:** 1 Oxford Eye Hospital, Oxford University Hospitals NHS Foundation Trust, Oxford, UK; 2 Nuffield Department of Clinical Neuroscience, University of Oxford, Oxford, UK

**Keywords:** Ocular gene therapy, Ocular inflammation, Uveitis, Clinical trials, Viral vectors

## Abstract

Retinal gene therapy has been shown to be a benefit in treating inherited retinal degenerations and may become the first line of treatment for patients with more common conditions such as age-related macular degeneration and diabetic retinopathy. A major limitation of ocular gene therapy is inflammation which has occurred in several clinical trials and may be the cause of dose limiting toxicity. Up until now it is been difficult to grade inflammation due to the small number of studies across many different centers. The absence of a standardized, reproducible system to grade retinal inflammation limits cross-study comparisons and consistent safety assessment. We synthesize evidence from published retinal gene therapy studies that report inflammatory outcomes and from our gene therapy center. Based on these findings, we propose a standardized grading framework—the vitritis, retinitis, microperimetry changes, fundus autofluorescence abnormalities, choroiditis, and subretinal infiltrates (VRMACS) score—to assess retinal inflammation over time using clinical examination and multimodal imaging. The system evaluates 6 domains: vitritis, retinitis, microperimetry changes, fundus autofluorescence abnormalities, choroiditis, and subretinal infiltrates. Grading allows for structured, longitudinal assessment of inflammatory severity after gene therapy. The VRMACS score provides a standardized grading system that may improve detection, quantification, and reporting of inflammation associated with subretinal ocular gene therapy. Standardized inflammation grading could support dose selection, guide prespecified stopping rules, and improve consistency in the interpretation of inflammation-related adverse events across early- and late-phase trials. From a regulatory perspective, adoption of this system may support consistent safety assessment across clinical trials and facilitate regulatory evaluation of inflammation-related adverse events. Clinically, VRMACS offers a practical tool for monitoring patients receiving retinal gene therapy and guiding timely intervention.

Ocular gene therapy using recombinant adeno-associated viral (AAV) vectors has emerged as a transformative approach for the treatment of inherited retinal and optic nerve diseases. By delivering functional copies of disease-causing genes to target cells, these therapies offer the potential to restore or preserve vision in disorders that would otherwise result in progressive visual loss. The eye is particularly well suited for gene therapy because of its small size, compartmentalized anatomy, relative immune privilege, and accessibility for localized delivery and longitudinal monitoring.

The regulatory approval of voretigene neparvovec for RPE65-associated Leber congenital amaurosis validated this therapeutic strategy and accelerated the development of gene therapy trials for other inherited retinal diseases, including choroideremia, X-linked retinitis pigmentosa (RPGR), X-linked retinoschisis, and Leber hereditary optic neuropathy.^1^ More recently, ocular gene therapy has expanded beyond rare inherited conditions, with AAV-based approaches increasingly being investigated for common retinal diseases such as age-related macular degeneration and diabetic retinopathy.^2^^,^^3^ This shift substantially broadens the potential patient population and underscores the importance of robust and standardized safety assessment.

Although many clinical trials have reported encouraging signals of efficacy, intraocular inflammation has emerged as the most frequently reported adverse event associated with ocular AAV gene therapy, particularly after intravitreal delivery.^4^ Despite its clinical and regulatory significance, inflammation is inconsistently defined and variably reported across studies.

We synthesize available clinical trial evidence and data from our gene therapy center to propose a structured framework—the vitritis, retinitis, microperimetry changes, fundus autofluorescence abnormalities, choroiditis, and subretinal infiltrates (VRMACS) system—for grading posterior segment inflammation based on clinical examination and characteristic imaging findings. This approach enables reproducible longitudinal assessment, supports differentiation of procedure-related and vector-mediated inflammation, and provides a standardized tool for clinical monitoring and safety evaluation in ocular gene therapy trials.

## Clinical Trial Evidence

### RPE65-Associated Retinal Dystrophy

Voretigene neparvovec (Luxturna) is an AAV2 vector delivering the RPE65 gene by subretinal injection and was the first ocular gene therapy approved for clinical use.^5^^,^^6^ In the pivotal phase III trial (n = 31) and extension studies, patients received 1.5 × 10^11^ vector genomes per eye with systemic corticosteroids.^5^ Among 37 treated participants, ocular inflammation was infrequent, with no detected immune responses and 1 case of endophthalmitis attributed to the surgical procedure. Visual improvements were sustained for ≥4 years, supporting a favorable safety profile. The low incidence of inflammation is attributed to the relatively small dose, subretinal delivery, fluid air exchange to remove refluxed vector particles, and perioperative steroid regimen.

Bainbridge and colleagues’ phase I–II, open-label study of subretinal rAAV2/2–RPE65 in 12 participants occupies a pivotal position in ocular gene therapy, providing both an efficacy signal and an early caution regarding assumptions of immune privilege.^7^ Approximately half of treated participants showed improved retinal sensitivity, peaking at 6 to 12 months and then declining, despite absent electroretinographic improvement and the occurrence of intraocular inflammation and vision loss in a subset. The lasting importance of this study lies less in the magnitude of functional gain than in its demonstration that subretinal AAV delivery can provoke clinically significant inflammation even with systemic corticosteroids, complicating interpretation of both safety and durability. Importantly, vector preparation in the early studies was less than adequate and this particular study had 10% full to empty capsid ratio on top of a very high subretinal dose of 10^12^ genome particles. Hence, the severe information seen in 3 of the 8 patients receiving the high dose will most likely not be repeated in studies going forward which limit the subretinal dose to around 10^11^ and with a >80% full to empty capsid ratio, which limits the overall viral load triggering information.^8^

Several trial design features limit causal inference. The study was small, heterogeneous in age, nonrandomized, and unmasked, with the fellow eye serving as a comparator. These constraints are understandable for early gene augmentation work but become problematic when endpoints (dark-adapted perimetry, microperimetry, and navigation tasks) are susceptible to learning effects, fixation instability, and ceiling/floor constraints typical of advanced retinal degeneration. Many standard visual function measures are insufficiently sensitive to detect subtle treatment effects, making prespecified reproducibility essential to avoid overinterpretation of noisy data.^9^ In this context, a transient improvement followed by decline may reflect biology (vector expression, immune attenuation, and disease progression), measurement variability, or fluctuating patient engagement, with inflammation adding further uncertainty.

The safety signal reported by Bainbridge and colleagues—3 participants with severe intraocular inflammation and 2 with clinically significant visual acuity loss—warrants detailed phenotyping rather than just an adverse event footnote.^7^ Grading of anterior chamber and vitreous activity, multimodal imaging correlates (OCT biomarkers such as hyperreflective foci, subretinal deposits, retinal or choroidal thickening, ellipsoid zone perturbation; widefield fundus autofluorescence patterns), and explicit documentation that differentiates inflammatory findings attributable to surgical manipulation (e.g. vitrectomy, retinotomy creation, and subretinal bleb formation) from those arising through vector-mediated immunogenic mechanisms, are required for accurate interpretation of safety signals and durability. Early trials often reported inflammation as a binary outcome despite management decisions depending on phenotype and severity and delayed recognition being particularly consequential in structurally fragile retinas.

Notably, subsequent synthesis of immunogenicity data around this trial complicates the apparently modest inflammatory incidence. Despite a 5-week tapering course of oral prednisolone starting 1 week before surgery, immune-related events occurred in 5 of the 8 high-dose participants (note that the capsid dose was in the region of 10^13^), including the development of anti-AAV2 neutralizing antibodies in two patients and a capsid-specific interferon gamma enzyme-linked immunosorbent spot response in 1 patient.^7^ In that enzyme-linked immunosorbent spot-positive participant, inflammation was associated with worsening visual function. First, these findings suggest that clinical inflammation may represent only part of the immune response that also includes subclinical humoral or cellular activation, responses that may still influence transgene expression and retinal homeostasis. Second, they indicate that prophylactic systemic steroids, while rational, are not uniformly protective; their timing, dose, and taper may suppress symptoms without eliminating immune priming, potentially delaying detection until function is affected. Hence, structured monitoring of clinical and imaging biomarkers is essential.

Finally, although the original investigators emphasized species differences in RPE65 requirements to explain limited durability, durability in ocular AAV therapy is better understood as a composite outcome shaped by transgene expression, surgical trauma, and immune-mediated effects. Modern practice anticipates immunologic risk, mandates perioperative steroids, and recognizes surgical variables (bleb geometry, foveal stress, and retinotomy placement) as contributors to structural change that may mimic or amplify inflammation.^10^ Together, these studies established early that inflammation is not merely a tolerability concern but a central determinant of monitoring, management, and interpretation of efficacy in ocular gene therapy.

### Choroideremia: Early Trials and REGENERATE Study

Early choroideremia studies using subretinal AAV2-REP1 established important precedents for understanding gene therapy-associated uveitis.^11^, ^12^, ^13^ In the phase I trial, 6 men with advanced choroideremia received subfoveal AAV2-REP1 with a 21-day tapering course of high-dose oral prednisone starting 2 days before surgery.^11^ Postoperative monitoring focused on inflammation in the anterior chamber, vitreous, and subretinal space. This approach largely prevented overt uveitis, supported by close surveillance for functional and imaging changes, including reduced microperimetry responses, cystoid macular oedema, and subretinal hyperreflective densities. However, immune-related complications still occurred, including 1 case of localized intraretinal inflammation with subretinal and vitreous hemorrhage. Thus, while early trials demonstrated favorable safety profiles and stable or improved visual acuity in some eyes, they also showed that vector- or surgery-related inflammation can cause tissue damage and may not be fully prevented by short courses of corticosteroids.

The open-label phase II REGENERATE trial further highlighted the clinical impact of inflammatory events.^14^ At 24 months, changes in best-corrected visual acuity did not differ significantly between treated and control eyes (–2.63 vs. + 2.67 ETDRS letters; *P* = 0.08), although treated eyes showed greater visual field loss, attributed in part to surgery.^14^ The finding of visual field loss underscores the difficulty of distinguishing immune-mediated injury from effects of retinotomy or subretinal bleb formation when inflammation is not defined by specific ocular compartments and adverse events are broadly categorized. Notably, differences in visual fields were no longer significant when analysis was restricted to eyes without inflammatory complications—retinal degeneration progressed similarly in treated and control eyes.^14^ These results emphasize the need for standardized surgical approaches and more precise inflammation classification to distinguish vector-related immune injury from surgical effects.

The STAR study, which enrolled 169 adult men, provided the largest data set for choroideremia gene therapy to date. Inflammation-related treatment-emergent adverse events occurred in 51% of high-dose eyes (1 × 10^11^ vector genomes) and 47% of low-dose eyes (1 × 10^10^ vector genomes) versus untreated controls.^15^ One participant in the high-dose group developed severe noninfective retinitis. Most inflammatory events clustered within the first 30 days and were attributed to surgery or transient vector reflux rather than dose.^15^ These findings indicate that ocular inflammation is common but generally manageable and highlight the challenge of consistent dose delivery with subretinal injection. Reliance on regulatory adverse event categories also grouped mild anterior inflammation with severe posterior disease, limiting mechanistic insight.

### Choroideremia: GEMINI Bilateral Sequential Administration

The GEMINI study attempts to answer a practical question: if subretinal AAV gene augmentation is intended to preserve binocular function, can we treat both eyes sequentially without provoking a deleterious fellow-eye effect from immune priming?^16^ In this multicenter, open-label, 2-period phase II safety study, 66 adult men with genetically confirmed choroideremia received bilateral subretinal timrepigene emparvovec (AAV2-REP1; 1 × 10^11^ vector genomes in up to 0.1 mL per eye), with the second eye treated after variable intervals (<6 months, 6–12 months, or >12 months) and each eye followed for 12 months.^17^ The study prioritized inflammatory endpoints and immunogenicity, reflecting recognition that ocular immune privilege is conditional and that bilateral treatment represents repeat antigen exposure.

Inflammatory events were frequent but generally manageable. Ocular inflammation-related treatment–emergent events occurred in 66.7% of participants (123 events), and retinal inflammation-related events in 45.5% (52 events).^17^ Anterior chamber inflammation was reported in 60.6% of participants and vitritis in 16.7%, with less frequent macular or cystoid macular oedema. Although these rates indicate that inflammation after subretinal AAV delivery is common, most events (95.5%) were responsive to corticosteroids, and not associated with clinically meaningful vision loss at 12 months.^17^ Serious inflammatory events were uncommon, with 2 participants (3.0%) developing noninfective retinitis. Importantly, inflammatory profiles were similar between first and second eyes, with no evidence of immune priming or increased risk after initial exposure.

Immunogenicity analyses provide additional context for bilateral treatment planning. Approximately one-third of participants were baseline positive for AAV2 neutralizing antibodies.^17^ Treatment–emergent antibody positivity peaked at month 1, while cellular responses peaked later. Although baseline antibody status was not associated with retinal inflammation, it was linked to higher rates of ocular inflammatory events and reduced visual acuity after first-eye treatment. In period 2, a ≥15-letter visual acuity loss occurred in 4 eyes among 3 participants with pre-existing antibodies, without a clear cause.^17^ These findings suggest that serostatus may not predict retinitis but may signal increased risk of postoperative inflammation or functional decline, supporting immune assessment and cautious counseling rather than exclusion.

GEMINI also highlights cumulative surgical risk. Five participants (7.6%) experienced serious procedure-related adverse events, including vitreous hemorrhage, macular hole, retinal detachment, or choroidal neovascularization.^17^ Although visual acuity was generally preserved, bilateral treatment doubles exposure to surgical risk in structurally fragile retinas.

Key strengths include the study’s scale, structured follow-up, and vector-shedding surveillance, which demonstrated transient systemic exposure resolving within weeks. Stratification by intersurgery interval further acknowledges the immunologic relevance of treatment spacing. Limitations include reliance on broad regulatory adverse event categories rather than standardized uveitis grading and limited evaluation of very short inter-eye intervals used in some approved protocols (e.g., voretigene neparvovec’s phase III design treated the second eye 6–18 days after the first).^5^

Overall, GEMINI supports the feasibility of bilateral sequential subretinal AAV delivery without clear evidence of increased immunologic risk to the second eye. Its principal contribution is methodological, underscoring the need to treat inflammation as a graded, compartment-specific process with objective imaging and prespecified management pathways.

### X-Linked Retinitis Pigmentosa: AAV8.coRPGR Trial

The XIRIUS phase I/II dose-escalation trial of subretinal AAV8.coRPGR for RPGR-associated X-linked retinitis pigmentosa is particularly informative because it treated inflammation as an expected, dose-dependent biological response rather than an incidental complication.^18^ Eighteen participants across 6 ascending dose cohorts received a standardized 21-day course of oral prednisolone beginning before surgery and were prospectively monitored for intraocular inflammation. Vector doses were calculated using linearized plasmid rather than supercoiled DNA as a reference standard, placing reported doses approximately half a log unit higher than those in earlier studies.^19^

The study met its prespecified safety endpoint at 6 months, with no serious adverse events or dose-limiting toxicities.^18^ Inflammation was absent at doses up to 5 × 10^11^ gp/mL (cohorts 1–3) but occurred in 7 of 9 participants at higher doses (cohorts 4–6), presenting as mild subretinal inflammation confined to the treated bleb.^18^ OCT showed hyperreflective subretinal deposits without vasculitis, cystoid macular edema, vitritis, or chorioretinal lesions.^18^ One case of anterior uveitis, considered postoperative, resolved with topical corticosteroids.^18^

The key finding was not the absence of inflammation but its predictable behavior: responses were dose related, anatomically confined, and consistently steroid responsive, resolving within approximately 6 months without additional immunosuppression. After subretinal AAV delivery, inflammation predominantly manifested as posterior changes, often accompanied by transient reductions in retinal sensitivity, and responded to timely corticosteroid treatment.

XIRIUS offers important methodological lessons. Baseline microperimetry was performed in triplicate to minimize learning effects, improving interpretability when used as both an efficacy measure and a functional marker of inflammation. In exemplar case (C4.1), reduced retinal sensitivity coincided with OCT-visible subretinal lesions and improved promptly with high-dose oral prednisone, demonstrating a treatable inflammatory process that could otherwise be mistaken for loss of treatment effect.^18^ These findings suggest that inflammation identified solely through imaging and microperimetry may be underestimated.

#### Neovascular Age-Related Macular Degeneration

Campochiaro et al reported the phase I/IIa dose–escalation study of subretinal RGX-314, an AAV8 vector encoding an anti-VEGF-A antigen-binding fragment, in 42 patients with neovascular age-related macular degeneration aged 50–89 years who had previously received anti-VEGF therapy. Participants received a single subretinal administration across 5 dose levels, ranging from 3 × 10^9^ to 2.5 × 10^11^ genome copies per eye. The authors concluded that subretinal RGX-314 was generally well tolerated, with no clinically recognized immune responses and no inflammation beyond that expected after routine vitrectomy. This study is therefore notable as it suggests that subretinal AAV-mediated anti-VEGF gene therapy can be delivered with a low clinically apparent inflammatory signal in an older neovascular age-related macular degeneration population. However, the study does not establish that older age itself is protective against inflammation. A more likely explanation is that tolerability may relate to features of subretinal delivery, including relative compartmentalization of vector exposure compared with intravitreal administration, vector dose selection, surgical technique, and perioperative management. Although immunosenescence could theoretically attenuate some adaptive immune responses in elderly patients, this remains speculative in ocular AAV therapy. Indeed, recent preclinical ocular AAV data reported increased and more persistent inflammation with age, arguing against a simple protective effect of older age.^20^

### Dose, Route of Administration, and Inflammatory Risk

Across trials, inflammatory risk varies with both vector dose and route of administration. Subretinal delivery confines the vector beneath the neurosensory retina, limiting systemic exposure and reducing immune activation. In the RPE65 program, 1.5 × 10^11^ vg delivered subretinally with systemic corticosteroids resulted in minimal inflammation, whereas in the phase I/IIa intravitreal AAV8-RS1 dose–escalation trial in X-linked retinoschisis, findings included anterior chamber activity, vitritis, optic disc hyperemia, and cystoid macular edema. These typically appeared 2 to 4 weeks postinjection and resolved with topical and/or oral corticosteroids without recurrence.^5^^,^^21^ Additionally, intravitreal AAV2 delivery for LHON exposed the entire vitreous cavity and led to inflammation in nearly 80% of treated eyes.^22^

Within subretinal trials, inflammation increased with dose. In RPGR-associated X-linked retinitis pigmentosa, inflammation was absent at doses ≤5 × 10^11^ vg but emerged at 5 × 10^12^ vg.^18^ Similarly, in X-linked retinoschisis, no inflammation occurred at 1 × 10^9^ vg, with dose-dependent events at 1 × 10^10^ and 1 × 10^11^ vg.^2^ In the phase III choroideremia trial, similar inflammation rates in high- and low-dose groups (51% and 47%) indicate that factors beyond total dose, including surgical reflux, contribute to inflammatory risk.^15^

Intravitreal delivery engages resident microglia and perivascular macrophages, while subretinal injection primarily exposes photoreceptors and retinal pigment epithelial cells.^23^ Pre-existing anti-AAV neutralizing antibodies may neutralize vectors in the vitreous, forming immune complexes that trigger complement.^24^ These observations support using the minimum effective dose and favor subretinal delivery when transducing the outer retina.

### VRMACS Classification

**Figure 1:**
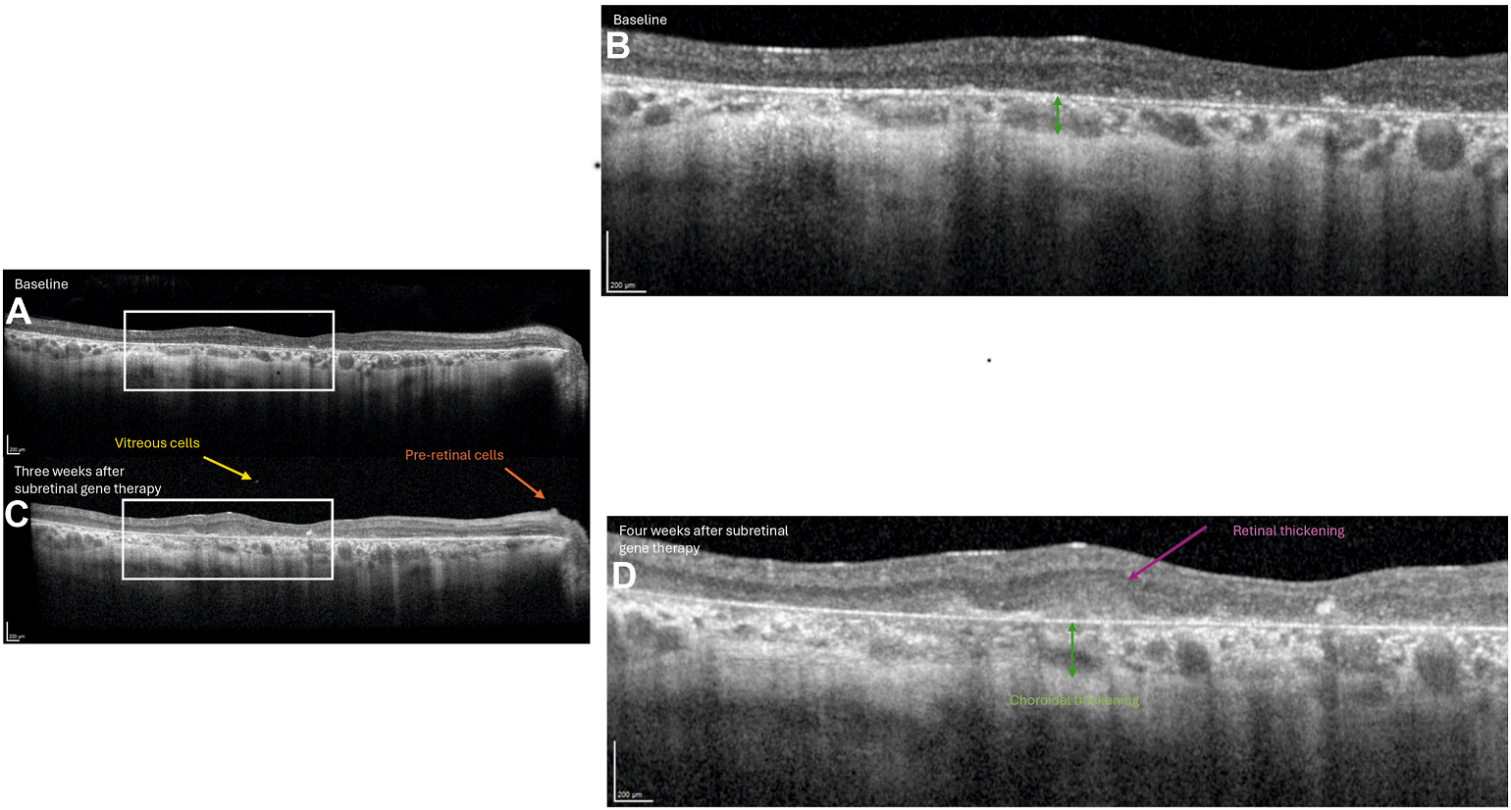
OCT demonstrating vitreous cells, preretinal deposits, retinal thickening, and choroidal thickening 4 weeks after subretinal gene therapy (**C** and **D**) compared to baseline (**A** and **B**).

**Figure 2:**
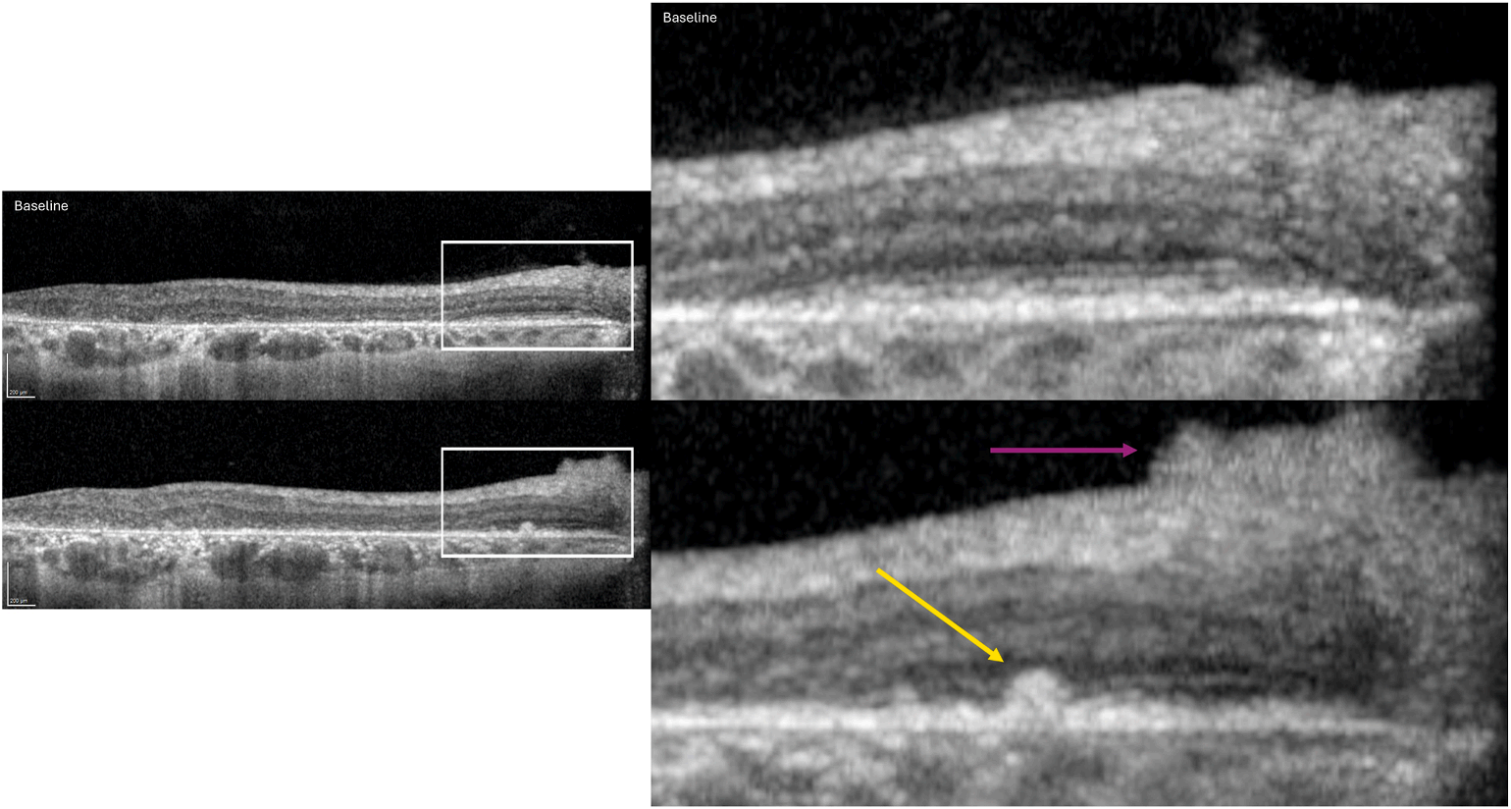
OCT demonstrating pre-retinal deposits and subretinal deposits—these tend to be triangular or dome-shaped elevations.

**Figure 3:**
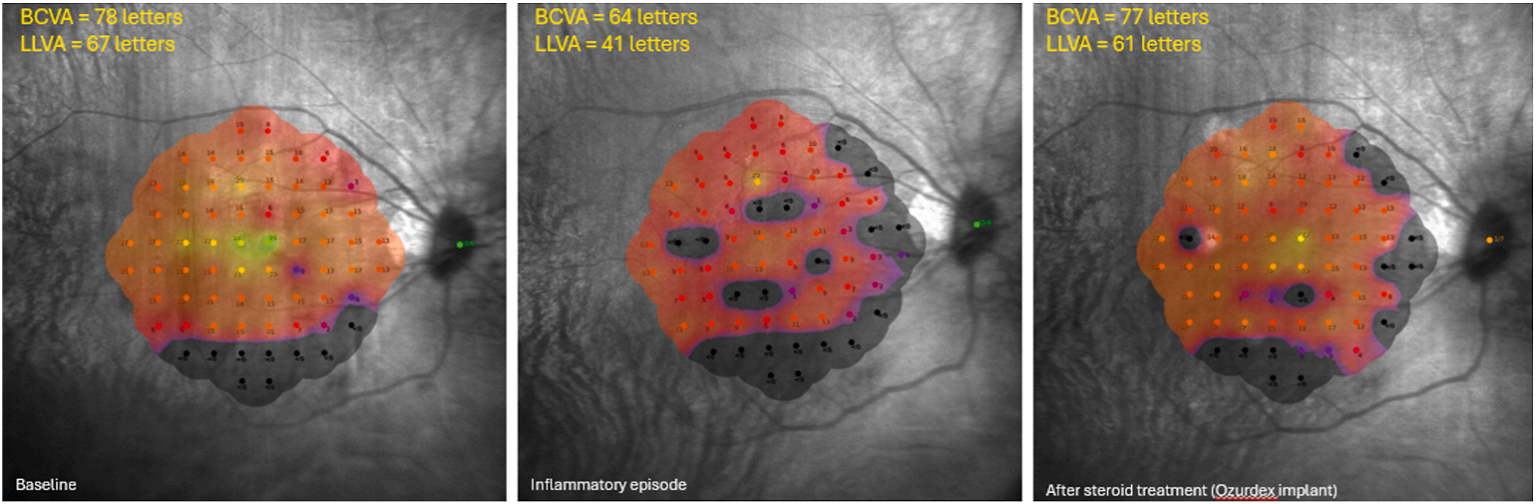
Reduced central retinal sensitivity in the treated eye, excluding the injections site, consequent to choroidal inflammation associated with the AAV8-coRPGR vector. There is a significant drop in LLVA compared to the decrease in BCVA. This effect subsides with steroid therapy. AAV = adeno-associated viral; BCVA = best corrected visual acuity; LLVA = low-luminance visual acuity.

Early detection of subclinical gene therapy–associated uveitis is vital (Table 1). Although vitritis and anterior or intermediate chamber activity may be visible on examination, OCT often detects inflammatory cells earlier (Figs 1 and 2). Posterior-predominant changes that may otherwise be occult include subretinal hyper-reflective deposits, retinal thickening or intraretinal fluid, and choroidal thickening, consistent with barrier disruption and inflammatory cell movement. Serial fundus autofluorescence provides complementary retinal pigment epithelium-level readout, helping distinguish inflammatory change from progressive degeneration when interpreted alongside OCT and clinical findings. Microperimetry offers an early, spatially resolved measure of inflammatory dysfunction and may precede irreversible structural damage, enabling timely escalation of corticosteroid therapy (Fig 3). Low-luminance visual acuity is a useful marker of early inflammation and central macular visual function before standard visual acuity declines.^28^

**Table 1:** Inflammatory Phenotypes Reported in Retinal AAV Gene Therapy

Sign (Clinical/Imaging)	Typical Compartment and Presentation	Proposed Pathophysiology	Implications for Monitoring/Management	References
Vitritis/vitreous haze (vitreous cells, haze; ± vasculitic leakage on FA)	Intermediate/posterior-predominant inflammation after intravitreal AAV; often subacute onset (weeks) with floaters, haze, or angiographic leakage	Innate and adaptive immune responses to capsid after intravitreal exposure (microglial activation, antigen presentation, lymphocyte trafficking) with breakdown of the blood–retinal barrier	Grade severity using SUN criteria; document FA leakage when suspected; monitor IOP. Escalate from topical to systemic corticosteroids when vitreous inflammation or vasculitis is present; treat complications (e.g., retinal tear) promptly	^21^
Subretinal/pre retinal hyperreflective “clumps/deposits” within bleb area on OCT	Posterior-segment, compartment-limited subretinal inflammatory phenotype after subretinal delivery; may coincide with transient functional regression	Localized inflammatory exudation and/or macrophage–RPE/photoreceptor debris aggregation in the subretinal space; may be dose-associated and influenced by surgical microtrauma at the bleb edge	Use OCT pattern recognition alongside function (microperimetry) to detect early; consider early oral steroid escalation when deposits emerge with sensitivity decline; distinguish from progressive atrophy by temporal evolution and steroid responsiveness	^18^
Change in fundus autofluorescence (FAF) signal/accelerated contraction of preserved FAF area	RPE-level disturbance (hyper-/hypo-autofluorescence) and/or progressive reduction in preserved FAF area; may be difficult to separate from natural history in slowly progressive disease	RPE stress and lipofuscin dynamics influenced by inflammation, surgical detachment/retinotomy, or natural degenerative progression; inflammatory injury may accelerate local RPE dysfunction and atrophy	Serial FAF with standardized acquisition; interpret alongside OCT and microperimetry. In REGENERATE, analyses in uncomplicated eyes emphasized that centripetal degeneration continued in both treated and control eyes, highlighting the need to attribute FAF change carefully.	^14^
Retinal thickening/intraretinal oedema (± cystoid change) on OCT	Inflammatory macular oedema phenotype; may be subtle initially but functionally meaningful in macula-targeted therapies	Cytokine-mediated vascular permeability and Müller cell dysfunction triggered by postoperative inflammation or vector-associated immune activation	Baseline and frequent early OCT; treat promptly with topical/periocular/systemic corticosteroids depending on severity and compartment; consider adjunctive therapy if oedema is recurrent or steroid-limiting	^21^^,^^25^
Choroidal thickening on OCT (± outer retinal “haze” or subretinal hyper-reflective material)	Posterior uveitis/choroiditis-like response; may accompany subretinal inflammation and correlate with subjective decline	Choroidal vascular hyperpermeability and inflammatory cell infiltration; may reflect posterior-segment immune activation rather than anterior segment reactivity	Measure subfoveal choroidal thickness longitudinally; combine with FAF and OCT biomarkers. Escalate systemic therapy if choroidal thickening accompanies functional deterioration or retinitis-like changes	^13^^,^^25^
Reduction in microperimetry sensitivity (transient or sustained)	Functional correlate that may precede obvious clinical uveitis; can localize to treated bleb footprint	Inflammation-driven photoreceptor dysfunction (oedema, cytokine toxicity, outer retinal stress) or evolving atrophy; transient reductions may improve with anti-inflammatory treatment	Use repeat baseline testing to reduce learning effects; monitor sensitivity maps over time. Trigger imaging review and steroid escalation when abrupt, localized sensitivity loss aligns with new OCT/FAF inflammatory features	^14^^,^^18^^,^^26^^,^^27^

AAV = adeno-associated viral; FA = fundus autofluorescence; IOP = intraocular pressure; RPE = retinal pigment epithelium; SUN = Standardization of Uveitis Nomenclature.

To address the lack of standardized reporting of posterior segment inflammation after subretinal gene therapy, we propose an OCT-based grading framework (Table 2). The framework specifies which structural and functional features are assessed, their anatomical location (vitreous, retina, subretinal space, and choroid), and thresholds for clinically meaningful change. This is based on analysis of previous clinical trials recognizing their heterogeneity (Table 3). Minimum reporting standards include documentation of onset, peak severity, resolution, compartmental involvement, and treatment response enabling consistent phenotyping, timely management, and cross-study comparison.

**Table 2:** Proposed OCT-Based Grading Framework for Intraocular Inflammation after Subretinal AAV Retinal Gene Therapy

Domain (Subretinal AAV Phenotype)	Definition and Measurement	Severity Grades (Anchored to Baseline)
Vitritis (V)	The presence of inflammatory cells within the vitreous cavity or on the retina.	V0: None V1: Vitreous cells or preretinal cells
Retinal thickening/intraretinal fluid (R)	Central subfield thickness (CST) change; report both percent change and foveal involvement.	R0: CST change <10%, no intraretinal fluid R1: CST change >10%
Decrease in microperimetry sensitivity (M)	Mean sensitivity change within treated area; minimize learning effects with repeat baseline testing;^25^^,^^28^ interpret abrupt, localized loss with concurrent OCT change as inflammatory.	F0: Within test–retest variability F1: Decline in treated region beyond expected variability
Hypoautofluorescence (A)	Hypoautofluorescent areas not attributable to disease progression or retinotomy sites.	A0: No new decrease in autofluorescence A1: New hypoautofluorescence
Choroidal thickening (C)	Subfoveal choroidal thickness; interpret as percent change from baseline and relative to fellow eye when available.	C0: <10% increase C1: >10% or diffuse thickening
Subretinal hyper-reflective deposits (S)	New subretinal hyper-reflective foci, often triangular or dome-shaped.	S0: None S1: Subretinal deposits

AAV = adeno-associated viral.

**Table 3:** A Summary of the Subretinal Gene Therapy Clinical Trials Analysed

Study	Indication	Vector	Vector Load/Dose	Prophylaxis/Treatment Protocol Reported
Campochiaro et al, 2024	Neovascular age-related macular degeneration	RGX-314/ABBV-RGX-314; AAV8 anti-VEGF-A Fab	3 × 10^9^, 1 × 10^10^, 6 × 10^10^, 1.6 × 10^11^, or 2.5 × 10^11^ genome copies/eye	Steroid protocol not reported.
Russell et al, 2017; Maguire et al, 2019	RPE65-associated inherited retinal dystrophy	Voretigene neparvovec; AAV2-hRPE65v2	1.5 × 10^11^ vg in 0.3 mL per eye	Oral prednisone 1 mg/kg/day, max 40 mg/day, beginning 3 days before first-eye injection, followed by taper.
Bainbridge et al, 2015	RPE65-associated LCA/retinal dystrophy	rAAV2/2-RPE65	High-dose group is ∼10^12^ genome particles	Five-week oral prednisolone taper starting 1 week before surgery; primary paper verification recommended before final submission.
MacLaren et al, 2014	Choroideremia	AAV2-REP1	Up to 1 × 10^10^ genome particles	21-day high-dose oral prednisone taper starting 2 days before surgery.
Lam et al, 2019	Choroideremia	AAV2-REP1	1.0 × 10^11^ vector particles	Steroid protocol not reported
Fischer et al, 2019	Choroideremia	AAV2-REP1	1.0 × 10^11^ vector particles	Steroid protocol not reported
Cehajic-Kapetanovic et al, 2024/REGENERATE	Choroideremia	AAV2-REP1	1.0 × 10^11^ vector particles	Steroid protocol not reported
MacLaren et al, 2023/STAR	Choroideremia	Timrepigene emparvovec/BIIB111; AAV2-REP1	High dose 1.0 × 10^11^ vg; low dose 1.0 × 10^10^ vg	21-day oral corticosteroid course beginning 2 days before dose.
MacLaren et al, 2024/GEMINI	Bilateral sequential choroideremia treatment	Timrepigene emparvovec/BIIB111; AAV2-REP1	1 × 10^11^ vector genomes in up to 0.1 mL per eye	The regimen consisted of 1 mg/kg/day, up to a maximum of 80 mg/day, from day 2 through day 7, followed by 0.5 mg/kg/day from day 8 to day 14, 0.25 mg/kg/day from day 15 to day 16, and 0.125 mg/kg/day from day 17 to day 18. For second-eye treatment, the full oral corticosteroid regimen was restarted 2 days before surgery if the second eye was treated on day 21 or later. If the second-eye surgery occurred before day 21, a new full regimen was started 2 days before the second-eye procedure and replaced the original taper.
Cehajic-Kapetanovic et al, 2020/XIRIUS	RPGR-associated X-linked retinitis pigmentosa	AAV8-coRPGR	Six ascending dose cohorts; manuscript states inflammation absent up to 5 × 10^11^ gp/mL and occurred at higher doses	21-day oral prednisolone course beginning before surgery; inflammation steroid-responsive and resolved without long-term additional immunosuppression.
Campochiaro et al, 2016	Neovascular age-related macular degeneration	Equine infectious anemia viral (EIAV) vector expressing endostatin and angiostatin under a CMV promoter	Three dose-ranging cohorts (2.4 × 10^4^ transduction units [TU], 2.4 × 10^5^ TU, and 8.0 × 10^5^ TU)	Steroid protocol not reported
Parker et al, 2022	ABCA4-associated Stargardt disease	EIAV-ABCA4 under a CMV promoter	The first 2 cohorts received the lowest dose, 1.8 × 10^5^ transduction units per eye. Cohort 3 received an intermediate dose of 6 × 10^5^ transduction units per eye. Cohorts 4 and 5 received the highest dose, 1.8 × 10^6^ transduction units per eye	No standardized systemic prednisolone regimen was reported. Perioperative corticosteroid use was left to the discretion of the surgeons and investigators. A combination of topical, peribulbar, or systemic corticosteroids was suggested, and all participants received topical corticosteroids for 2–6 weeks. In addition, on the day of surgery, 4 participants received 10 mg intravenous dexamethasone, 4 received a 10 mg periocular dexamethasone injection, and 1 participant received both intravenous and periocular corticosteroids.

AAV = adeno-associated viral; LCA = leber congenital amaurosis.

## Conclusion

The VRMACS grading system provides a useful reproducible and reliable method for quantifying inflammation associated with subretinal gene therapy. Having an objective measurement of this known complication is likely to help in the management of patients in future clinical trials and in managing treatment effects in gene therapies that are approved by the regulatory authorities.
